# A Multidisciplinary Intervention Utilizing Virtual Communication Tools to Reduce Health Disparities: A Pilot Randomized Controlled Trial

**DOI:** 10.3390/ijerph13010031

**Published:** 2015-12-22

**Authors:** John F. Emerson, Madelyn Welch, Whitney E. Rossman, Stephen Carek, Thomas Ludden, Megan Templin, Charity G. Moore, Hazel Tapp, Michael Dulin, Andrew McWilliams

**Affiliations:** 1 Department of Family Medicine, Carolinas HealthCare System, 2001 Vail Ave. Charlotte, NC 28205, USA; Madelyn.Welch@carolinas.org (M.W.); Stephen.Carek@carolinas.org (S.C.); Tom.Ludden@carolinas.org (T.L.); Hazel.Tapp@carolinas.org (H.T.); Michael.Dulin@carolinas.org (M.D.); Andrew.McWilliams@carolinas.org (A.M.); 2 Dickson Advanced Analytics, 720 E. Morehead Street, Charlotte, NC 28202, USA; Whitney.Rossman@carolinashealthcare.org (W.E.R.); Megan.Templin@carolinas.org (M.T.); Charity.Patterson@carolinashealthcare.org (C.G.M.)

**Keywords:** population health, virtual care, risk stratification, refractory to primary care, readiness to change, health coach

## Abstract

Advances in technology are likely to provide new approaches to address healthcare disparities for high-risk populations. This study explores the feasibility of a new approach to health disparities research using a multidisciplinary intervention and advanced communication technology to improve patient access to care and chronic disease management. A high-risk cohort of uninsured, poorly-controlled diabetic patients was identified then randomized pre-consent with stratification by geographic region to receive either the intervention or usual care. Prior to enrollment, participants were screened for readiness to make a behavioral change. The primary outcome was the feasibility of protocol implementation, and secondary outcomes included the use of patient-centered medical home (PCMH) services and markers of chronic disease control. The intervention included a standardized needs assessment, individualized care plan, intensive management by a multidisciplinary team, including health coach-facilitated virtual visits, and the use of a cloud-based glucose monitoring system. One-hundred twenty-seven high-risk, potentially eligible participants were randomized. Sixty-one met eligibility criteria after an in-depth review. Due to limited resources and time for the pilot, we only attempted to contact 36 participants. Of these, we successfully reached 20 (32%) by phone and conducted a readiness to change screen. Ten participants screened in as ready to change and were enrolled, while the remaining 10 were not ready to change. Eight enrolled participants completed the final three-month follow-up. Intervention feasibility was demonstrated through successful implementation of 13 out of 14 health coach-facilitated virtual visits, and 100% of participants indicated that they would recommend the intervention to a friend. Protocol feasibility was demonstrated as eight of 10 participants completed the entire study protocol. At the end of the three-month intervention, participants had a median of nine total documented contacts with PCMH providers compared to four in the control group. Three intervention and two control participants had controlled diabetes (hemoglobin A1C <9%). Multidisciplinary care that utilizes health coach-facilitated virtual visits is an intervention that could increase access to intensive primary care services in a vulnerable population. The methods tested are feasible and should be tested in a pragmatic randomized controlled trial to evaluate the impact on patient-relevant outcomes across multiple chronic diseases.

## 1. Introduction

Primary care delivery is undergoing a fundamental transformation as healthcare systems adapt to meet the triple aim of delivering high quality, patient-centered, cost-conscious care at the population level [1,2]. As the paradigm of care reimbursement shifts from quantity towards quality, healthcare systems and individual practices are grappling with how to effectively deploy population health strategies while meeting related requirements, like the patient-centered medical home (PCMH) model, which provides a framework for coordinated care delivery [3,4].

One intention of today’s policies driving healthcare change is to close the gaps in health disparities. These gaps have persisted in part because traditional models struggle to effectively deliver care to populations adversely affected by social determinants of health [5,6]. Barriers to healthcare access can result in inconsistent follow-up, which has in turn been correlated with poorly-controlled chronic medical conditions [7]. For example, diabetes mellitus type 2 is a chronic disease that has reached epidemic proportions with an estimated 29.1 million people affected in the U.S. and a total cost of $245 billion USD in 2012 [8]. In Mecklenburg County and North Carolina, diabetes rates are higher than national averages of age-adjusted diagnosed cases (8.9% and 10.2% *vs.* 6.9% in 2011) [9]. As a disease that requires close monitoring and frequent medication changes to prevent complications, achieving disease control can be particularly difficult for vulnerable populations. In general, traditional barriers, like geography, transportation and cost, not only affect a patient’s ability to access care, but also uniquely challenge a care delivery system’s ability to effectively provide population health outreach. Thus, as systems look to proactively engage vulnerable populations, innovative care delivery approaches are needed to cost-effectively improve access and provide the coordinated care called for in the triple aim.

Two promising solutions that may cost-effectively improve access and care coordination are the integration of telemedicine technology and health coaches into care delivery teams. First, telemedicine has been proposed as a mechanism to address the issues of access, quality and cost containment, because it potentially allows for centralized services to be delivered more conveniently to patients [10,11]. Incorporating telemedicine (including interventions, such as phone calls, teleconferencing, online glucose monitoring and web-based care management systems) as tools for chronic disease management has been studied with conflicting and inconclusive results [10,11]; however, diabetes care that is enhanced by telemedicine has shown an overall positive impact on surrogate markers of disease control, such as A1C [12,13,14]. Second, the use of health coaches to help connect patients to resources and motivate health changes is another approach that is gaining traction with a growing body of supporting evidence. For example, the implementation of a network of health coaches working in safety-net clinics [15,16,17,18] or engaged within a multi-disciplinary team in delivering care at the community level has been modeled and shown to be feasible [19]. The utilization of health coaches has also demonstrated significant improvements in several measures, including access to care, health literacy and accountability [15], as well as improving patients’ trust in physicians and medication adherence [16,17,18]. However, to our knowledge, the synergy of integrating health coaches and telemedicine technologies into the population health outreach efforts of a PCMH team has not been evaluated.

To capitalize on these types of promising interventions, healthcare systems must also develop strategies that identify patients at risk for poor outcomes and match these patients to the most effective outreach interventions. Current algorithms use the wealth of data from electronic health records and claims data to define patient cohorts at risk for variables like high costs [20], poor disease outcomes [21] or death [22,23]. However, it remains difficult to identify which specific patients within an identified “high-risk” group will benefit most from a resource-intensive outreach intervention. While measures exist that help define a patient’s readiness to make behavioral change, these measures have not been routinely linked to risk stratification and population health outreach. Previous research has demonstrated that identifying patients willing to utilize an integrative health model with established health and wellness goals resulted in improved patient activation, psychosocial measures and, ultimately, disease-related outcomes [24,25]. Specifically for diabetic patients, identifying individuals who are ready to make behavioral changes, such as improving diet, exercise and medication adherence, can predict improved glycemic control [24,25].

This pilot study aims to test the incorporation of telemedicine tools and health coaches into a population health outreach intervention for a vulnerable diabetic population with expressed readiness to make behavioral changes.

## 2. Experimental Section

### 2.1. Trial Design

This study is a pilot of a two-arm pragmatic randomized controlled trial of a multidisciplinary intervention utilizing advanced technology to improve chronic disease management in a vulnerable diabetic population. The institutional review board at Carolinas Healthcare System (CHS) approved the study protocol.

### 2.2. Participants

Eligible participants were uninsured patients between the ages of 18 and 75 years, living in four geographically-defined regions in Mecklenburg County, North Carolina, and with utilization at one of two CHS safety-net clinics over the prior 18 months. As a part of its mission to serve the community’s needs, CHS provides care to a large percentage of the uninsured population in Mecklenburg County. Patients are primarily cared for by a system of six safety-net clinics, including the two participating in this study. The clinics are not Federally-Qualified Health Centers, but are supported entirely by CHS and offer an income-eligible sliding-scale program for uninsured patients. Using a novel risk stratification algorithm (described below), patients were further selected based on poorly-controlled diabetes without a trend towards improvement or with inadequate primary care follow-up (Table 1). To more appropriately match resource-intensive population management to the patients most likely to benefit, patients were contacted via phone and asked a three-question screening survey to assess “readiness to change.” Participants were asked the following series of questions: (i) Do you consider yourself healthy (No = 1 point)? (ii) Are there any things you would like to change about your health (Yes = 1 point)? (iii) Can you give an example of how you or a healthcare provider could help you change your health for the better (Yes = 1 point)? To be screened in as ready to change and be eligible for enrollment, patients had to answer “no” to Question 1 and “yes” to Questions 2 and 3.

**Table 1:** Patient baseline demographics and comorbid conditions. GAD, generalized anxiety disorder.

		Intervention	Control	RPC * Target	RPC * Excluded After Review	RPC * Included After Review	RPC * Ready	RPC * Not Ready	RPC * Not Contacted
		*n* = 5	*n* = 5	*n* = 127	*n* = 66	*n* = 61	*n* = 10	*n* = 10	*n* = 41
Age	Years (mean)	47.6	48.8	46.7	48.0	45.4	48.2	42.7	45.3
Sex	Male	60.0%	60.0%	52.8%	53.0%	52.5%	60.0%	60.0%	48.8%
Race	Caucasian	20.0%	20.0%	14.2%	18.2%	9.8%	20.0%	0.0%	9.8%
	African American	60.0%	80.0%	61.4%	43.9%	80.3%	70.0%	90.0%	80.5%
	Other	20.0%	0.0%	24.4%	37.9%	9.8%	10.0%	10.0%	9.8%
Ethnicity	Hispanic	20.0%	0.0%	19.7%	28.8%	9.8%	10.0%	10.0%	9.8%
	Non-Hispanic	80.0%	100.0%	79.5%	69.7%	90.2%	90.0%	90.0%	90.2%
	Unknown	0.0%	0.0%	0.8%	1.5%	0.0%	0.0%	0.0%	0.0%
Comorbidities	Hypertension	80.0%	60.0%	64.6%	68.2%	60.7%	70.0%	40.0%	63.4%
	Hyperlipidemia	40.0%	0.0%	44.1%	51.5%	36.1%	20.0%	40.0%	39.0%
	Ischemic Vascular	0.0%	0.0%	6.3%	9.1%	3.3%	0.0%	10.0%	2.4%
	Depression/GAD	0.0%	0.0%	14.2%	15.2%	13.1%	0.0%	10.0%	17.1%
HgbA1c	mean	10.6	10.8	11.1	10.9	11.4	10.7	11.6	11.5

* RPC = refractory to primary care.

Patients were excluded if they were non-English speaking, established care with a different primary care clinic, obtained health insurance or had a late-stage terminal illness after a review of the electronic medical record by the principal investigator prior to being contacted. Patients were also excluded if upon being contacted, they were unable to come in for intake and exit interviews.

**Figure 1:**
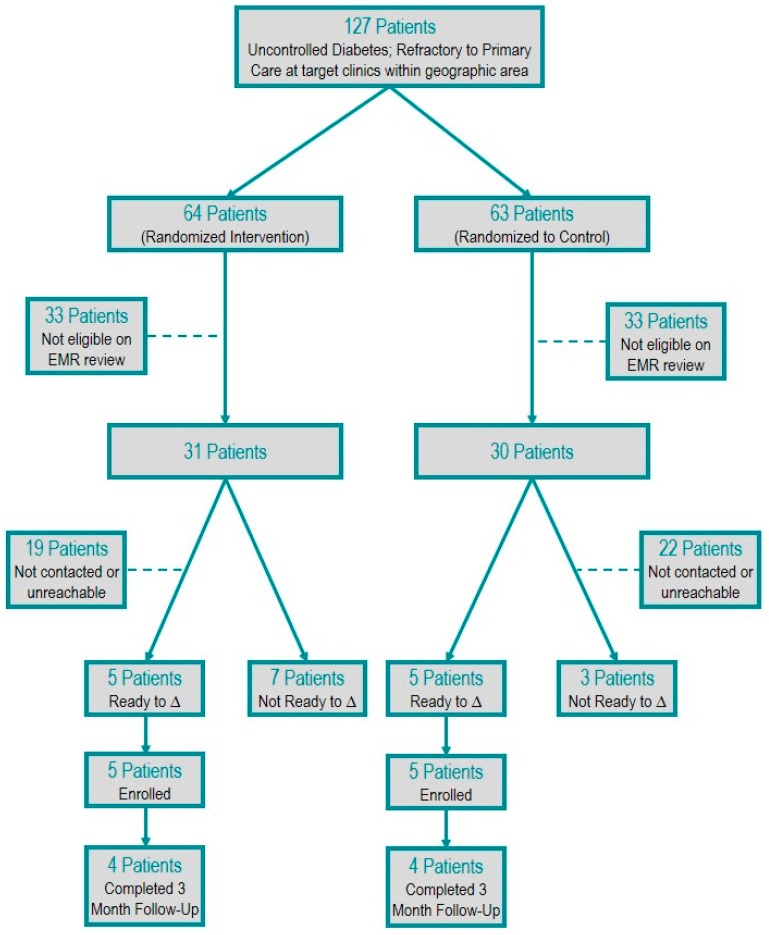
Patient selection flow diagram.

One-hundred twenty-seven high-risk, potentially eligible participants were randomized. Sixty-one met eligibility criteria, and 36 had contact attempted. Twenty-five patients had no contact attempts due to the resource limitations of the study personnel. Of the 36 patients for whom contact was attempted, 16 patients were unable to be reached after three attempts and, thus, were excluded. Twenty patients (32%) were contacted and screened for readiness to change. Of the 20 patients contacted, 10 patients screened out on the readiness to change survey, and 10 patients screened in and were enrolled. Eight of the enrolled patients completed the final three-month follow-up (Figure 1). 

### 2.3. Risk Stratification

Participants were defined as high risk using a novel algorithm intended to identify diabetic patients who failed to show improvement in the traditional outpatient primary care setting, called the “refractory to primary care (RPC) model.” Rather than looking at a cross-section of data to identify chronic disease risk, the RPC model examines patient data over time and incorporates the following criteria: (1) most recent documented hemoglobin A1C greater than or equal to 9; and (2) failure to show improvement of at least 20% in hemoglobin A1C over time or (3) failure to access recommended follow-up with no visit in the preceding 6 months.

### 2.4. Intervention and Comparison

Participants randomized to the intervention group received a 3-month (range 83–97 days) intensive multidisciplinary primary care intervention utilizing health coach-facilitated virtual visits and cloud-based glucose monitoring, called Carolinas Partners. Participants in both the control and intervention group continued to receive their usual care, which may or may not include other existing population health outreach interventions, primary care and specialty physician care services, behavioral health services and social services. All participants attended an initial and final in-person visit to measure hemoglobin A1C, blood pressure and psychosocial parameters through surveys.

### 2.5. Intensive Primary Care Intervention

The Carolinas Partners intervention provides intensive primary care services over a 3-month period. A health coach served as the only in-person contact for participants and facilitated communication, including virtual access to a multidisciplinary team consisting of a primary care physician, social worker, pharmacist and behavioral therapist. Additionally, all Carolinas Partners participants were given a cloud-based glucose monitor and testing strips and trained on how to use the devices at their intake visit. Each monitor functioned independently on cellular networks without the need for participant Internet connection.

Each intervention participant underwent an initial needs assessment that included goal setting, medicine reconciliation and evaluation by a clinical social worker, pharmacist and primary care provider to create an individualized 3-month care plan. The health coach attempted to contact participants at least once weekly by phone to provide ongoing diabetes education and reinforcement of the care plan. When necessary, the health coach facilitated real-time, tablet-based, virtual visits from participants’ homes to connect with providers on the multidisciplinary team. The primary care physician monitored blood glucose values several times a week through the cloud-based glucometers’ online system and also attempted outreach phone calls to the patients weekly or more frequently based on the assessed need. The primary physician contact for the project was a family medicine research fellow with time built into the academic schedule to allow such frequent contact, but this time was otherwise uncompensated. Ancillary staff utilized time outside of dedicated clinical hours to complete their respective components of the intervention. The health coach’s time was compensated for three half days weekly through the Department of Clinical Care Management. The multidisciplinary team met biweekly to discuss participant care plans and had on-going communication as needed through a secured intranet site that contained patient information on patient dashboards.

Communication was maintained between the participant’s established primary care physician and team through forwarding of pertinent clinical documentation in the electronic medical record and messaging as needed. At the conclusion of the intervention, a summary was created for each intervention patient and forwarded to the primary care provider for continuity purposes.

### 2.6. Outcomes

The primary outcome was assessment of both the feasibility of the protocol implementation and the operational logistics of the Carolinas Partners intervention in order to inform future studies. Protocol feasibility was evaluated in the areas of: risk model deployment, patient recruitment and enrollment, and study completion rate. The feasibility of the intervention was evaluated by: percent of successful virtual visits (defined by the ability to coordinate a tablet-based virtual visit with a health coach in the patient home), participant utilization of cloud-based glucometers and participant satisfaction based on post-participation surveys. Our primary efficacy outcome was meaningful access to PCMH services. Services were defined as any documented contact, including in person, phone or virtual, between a participant and a PCMH team member when a patient’s health or social situation was addressed. PCMH team members were defined as physicians, nursing staff, social workers, pharmacists, behavioral health counselors and health coaches. We chose to make access to PCMH services a primary efficacy outcome because easily-accessible care is a barrier commonly faced by vulnerable populations and also a necessity to improve poorly-controlled chronic disease. Examining PCMH access in this way allows the research team to better understand the potential for leveraging virtual care to overcome traditional barriers, like transportation, visit wait times and taking time off of work. Patient contact with PCMH services data was collected at the conclusion of the study by a review of the electronic medical record for both groups and by a review of the team-based intranet site, where contact with patients was documented for the intervention group.

Secondary outcomes included hemoglobin A1C control (<9%) and psychosocial assessments, which were evaluated at study entry and 3-month follow up for all participants. Hemoglobin A1C was measured using a point of care hemoglobin A1C machine, and blood pressures were recorded as the average of the three readings on an automated cuff. Patients completed quantitative surveys, including the Patient Health Questionnaire 9-item scale (PHQ-9), the Generalized Anxiety Disorder 7-item scale (GAD-7), the Patient Activation Measure 13-item scale (PAM-13) and the Veterans Rand 12-item health survey (VR-12) during intake and exit visits [22,26,27,28].

### 2.7. Sample Size

This project was designed to demonstrate the feasibility of conducting the described randomized controlled trial protocol, which could be achieved with relatively small sample sizes and without statistical analysis. Power analysis was conducted to determine significance regarding PCMH access, which was the primary efficacy outcome. In order to show a statistically-significant (*p* < 0.05) 50% relative difference in access to PCMH services defined by the mean number of visits, 20 patients would be needed in each study arm. At the time of the study design, we intended to aim for this enrollment target; however, due to an unexpected decrease in the personnel time allocated to the project and a delayed start to the project, we were forced to decrease our target enrollment from 40 patients to 10. As a consequence of the small sample size, no statistical analysis of the primary efficacy outcome is provided.

### 2.8. Randomization

**Figure 2:**
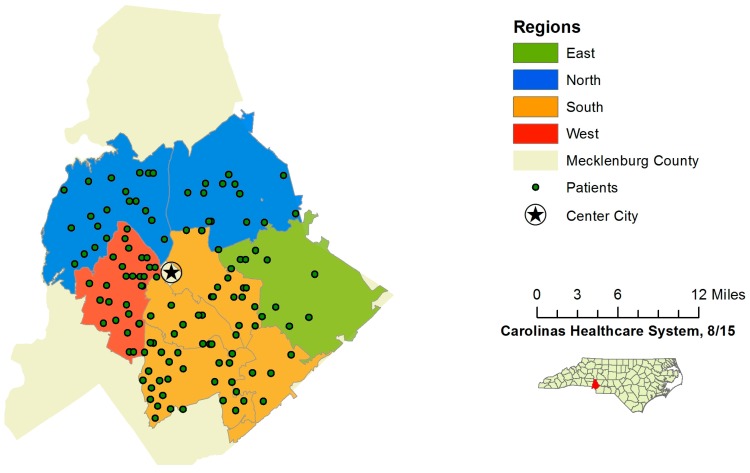
Region map of Mecklenburg County, NC.

All patients meeting the initial inclusion criteria after a query of the data available through the electronic medical record data warehouse underwent pre-consent randomization to either control or intervention groups. These groups were then stratified into four geographically-defined regions within Mecklenburg County. Randomization was completed by the data analytics department at Carolinas Healthcare System, and study personnel were not involved in the randomization process. Regions were included in the randomization to demonstrate the method of tailoring future interventions aimed at health disparities at the neighborhood level, a component that would be vital in a larger study. After randomization, all patients’ charts were reviewed in depth to assess exclusion criteria. Patients who were still eligible after chart review were then contacted by phone to conduct the “readiness to change” screening survey in sequential order, starting in regions east, north and south. Due to limited resources, patients in the west region were not contacted (Figure 2). Pre-consent randomization was chosen for this pilot study, because we wished to demonstrate the feasibility of using this method for a larger pragmatic trial. A larger such trial would be designed as a randomized quality improvement study where the entire “at-risk population” would be randomly assigned to usual population health management or a minimal risk intervention, like that studied here. The exclusion of patients after randomization does introduce bias, but in a pragmatic trial, this would translate to a clinically-relevant step taken by clinical staff to confirm eligibility for a quality improvement program. Ideally, the process is part of routine care delivery and workflows. Indeed, because of the clinical equipoise between interventions, Pletcher and colleagues even suggest that trials like these be considered for exceptions to informed consent [29]. While that may be true for a larger study, we were performing this pilot on a very small scale and, thus, felt participant consent was necessary. We received a waiver of documentation of consent. Additionally, this randomization method enables a pragmatic trial design to stratify patients by geographic area, allowing assignment of health coaches at the neighborhood level. Again, this was not relevant for a small pilot, but in a larger trial, the health coaches would ideally be hired from the specific communities in which they will work. Cluster randomization could be considered as an alternative method; however, we wanted to keep the intervention and eventual analysis at the patient level.

## 3. Statistics

Descriptive statistics, including counts and percentages, were reported for baseline and demographic variables, feasibility measures and outcomes. The frequency of each type of PCMH service was counted for each participant. Initial and final hemoglobin A1C values and survey results were compared by patient. Survey results were also graphed by patient and treatment groups.

## 4. Results

In total, 13 of 14 attempted virtual visits were deemed successful based on the ability to connect and conduct a health management visit. These visits were all completed in patient homes with the assistance of a health coach, who set up a tablet device and established a connection using a 4G hot-spot. One visit had technical difficulties with establishing a connection initially, but a connection was established within a few minutes, and the remainder of the visit was successful. One patient was not in the home at the time the health coach arrived for a scheduled virtual visit, and this was the only unsuccessful visit. Three of the five intervention participants utilized their cloud-based glucometer regularly with 50 or more data points over the course of the three months (range 4–136). There were 31 phone contacts with patients for PCMH services in the intervention group; however, some patients were unreachable on multiple attempts to coordinate services or discuss medical management. All four of the intervention participants, who completed the study, indicated that they would recommend this type of intervention to a friend, and three out of four felt that the intervention improved their overall health. Three out of four also indicated their willingness to use virtual care visits to help manage their health in the future if available. 

**Figure 3:**
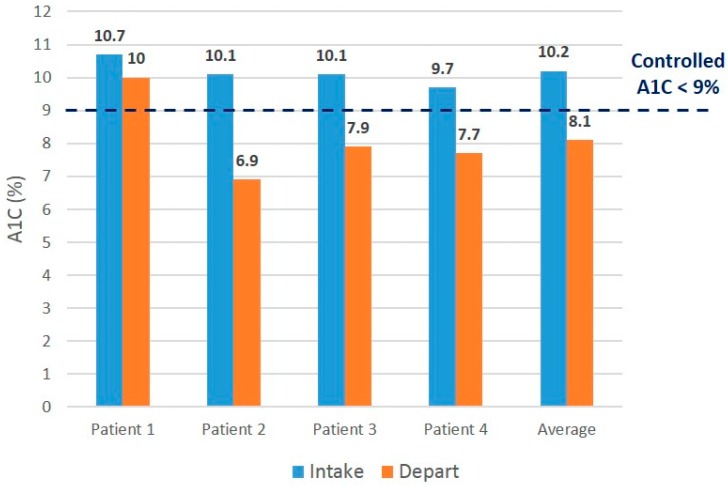
Initial *vs.* final Hgb A1C values for intervention patients.

**Figure 4:**
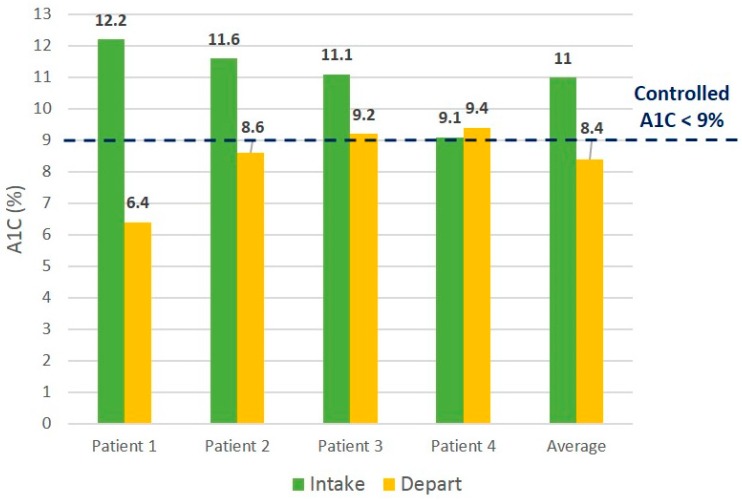
Initial *vs.* final Hgb A1C values for control patients.

The intervention patients accessed more PCMH services compared to the control patients (median nine points of access *vs.* four points), as well as more PCMH phone contact (median three phone contacts *vs.* zero phone contacts) (Table 2). All participants in the intervention group showed improved hemoglobin A1C values, and three out of four control participants showed improved values (Figure 3 and Figure 4). Three out of four intervention participants and two out of four control participants demonstrated post-hemoglobin A1C values meeting the *a priori* definition of “not poorly controlled” or hemoglobin A1C of less than 9%.

There were also anecdotal wins in the intervention group. For example, one intervention patient was unable to afford the necessary co-pay for visits, thus limiting access to care. A simple medical outreach intervention would have failed to help him navigate access to necessary pharmacotherapy and follow-up. However, with this intervention, the team’s social work assessment helped place the patient into a system-based assistance program, ultimately allowing him to re-establish regular care and afford much needed medications.

**Table 2:** Meaningful access to patient-centered medical home (PCMH) services over 3 months.

Carolinas Partners (Intervention Group)	Total PCMH Access*	PCMH Office Visits	PCMH Phone Contact	PCMH Virtual Visits
Patient 1	14	1	10	3
Patient 2	21	3	15	3
Patient 3	9	2	3	4
Patient 4	8	2	3	3
Patient 5	1	1	0	0
Carolinas Partners Median:	9	2	3	3
**Standard Care (Control Group)**	Total PCMH Access*	**PCMH Office Visits**	**PCMH Phone Contact**	**PCMH Virtual Visits**
Patient 1	4	3	1	*N/A*
Patient 2	6	3	3	*N/A*
Patient 3	0	0	0	*N/A*
Patient 4	5	5	0	*N/A*
Patient 5	0	0	0	*N/A*
Standard Care Median:	4	3	0	*N/A*

* Meaningful access to PCMH defined as any type of in-person office visit, phone conversation or virtual visit conducted by primary providers clinic and staff (PCMH) at which time assessment and management of health-related conditions was conducted by a healthcare provider (physician, nursing staff, social worker, pharmacy, behavioral health and health coach).

Of the 10 patients who were enrolled, two patients withdrew, one each in the control and intervention group. The intervention participant was unable to be contacted on multiple attempts via phone following his initial presentation for enrollment, and it was later learned he moved to another state. The control participant asked to no longer be contacted shortly following enrollment due to a family member’s change in health status.

**Figure 5:**
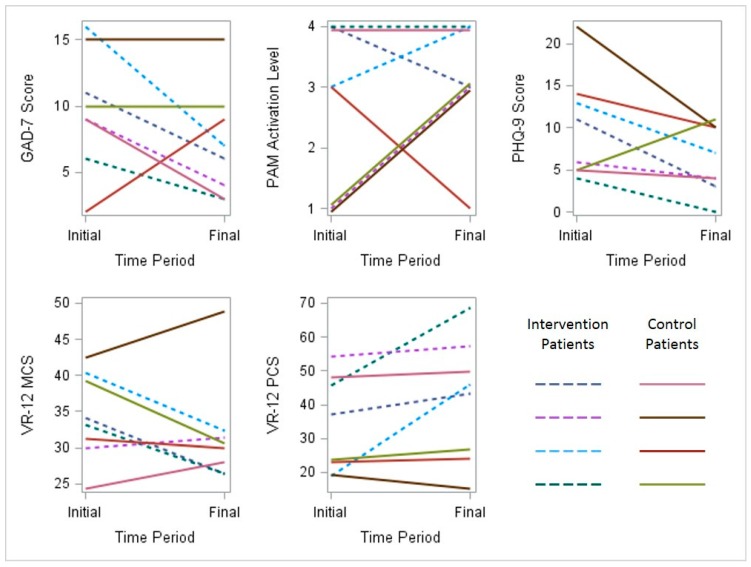
Initial and final behavioral health survey results for all participants. PAM, Patient Activation Measure; PHQ, Patient Health Questionnaire; VR, Veterans Rand (MCS, Mental Component Score) (PCS, Physical Component Score).

Scores for depression, anxiety, activation and perceived quality of life were documented for participants in both groups at the time of enrollment and the completion of the study. This limited dataset is described in graphical form (Figure 5).

GAD-7 measures generalized anxiety symptoms on a scale from zero (no symptoms) to 21 (severe anxiety symptoms). A score of seven or greater is generally considered clinically significant. PAM-13 measures respondents’ levels of activation and engagement in their personal health. Score ranges are grouped into four levels in order of increasing ability and confidence in managing healthy behaviors: (1) disengaged and overwhelmed; (2) becoming aware, but still struggling; (3) taking action; and (4) maintaining behaviors and pushing further. PHQ-9 measures the presence of depressive symptoms ranging from zero (no symptoms) to 27 (severe depressive symptoms). A score of 10 or greater is generally considered clinically significant. VR-12 measures general health-related quality of life perceptions for both mental components and physical components , with higher scores indicating better health status.

## 5. Discussion

This randomized controlled trial was designed to test the feasibility of a pragmatic and innovative population health outreach intervention in uninsured diabetic patients. The pilot study demonstrates the feasibility of a number of key components that set the stage for a larger trial. First, we demonstrated the necessary steps for conducting a pragmatic randomized controlled trail of a complex, intensive primary care intervention that can be both tailored to and studied at the level of a patient’s community. Furthermore, the data generated on patient eligibility, accessibility and retention will inform future study design and power analyses. Second, we successfully incorporated telemedicine and health coaches into an intervention serving a vulnerable population. Third, we showed the ability to incorporate readiness to change into a novel method of risk stratification for population health outreach. As healthcare systems adopt population health strategies, increased demand for evidence supporting the medical and cost effectiveness of interventions will be needed. This study will help inform the design of pragmatic trials to evaluate population health interventions and offer descriptions of intervention components that warrant further study.

In developing an evaluative approach for defining patients as “refractory” to traditional primary care, the primary goal was to intervene when the patient demonstrated a failure to improve or was not receiving standard care according to disease severity earlier in the disease process in the hopes of preventing disease progression and morbidity. Although there are morbidity indexes and risk assessment tools to define patient risk, to our knowledge, none assess risk of poor outcomes utilizing longitudinal data demonstrating lack of care or improvement over time.

The readiness to change questionnaire was developed after lessons learned from prior pilot studies. These previous pilots provided patients with similar resources, but ultimately, some patients were disengaged and unwilling to collaborate with a shared goal of positive behavioral change. Although readiness to change has been well studied and validated in alcohol cessation research [30], utilizing these techniques to determine appropriateness for population health interventions in chronic disease care has not been utilized to our knowledge. This short readiness to change survey draws upon motivational interviewing and behavioral change techniques to identify patients with insight into their disease severity and a desire to change while maintaining scalability. The improvements in hemoglobin A1C seen in both our intervention and control patients suggest the potential merit of adding readiness to change screening to standard risk stratification measures. To be scalable, a survey like this must be brief and preferably able to be administered by non-clinical staff.

The use of multidisciplinary services, health coaches and advanced technology, including virtual visits and cloud-based glucometers, has previously demonstrated success in improving care delivery, but no studies have examined a physician-led team that reaches out to a vulnerable population through a virtual platform facilitated by a health coach. The virtual care component of this model expands the reach of a centrally-located care team and overcomes many traditional access barriers, while the incorporation of health coaches allows the team to be tailored to the unique needs of individual communities. Other studies have assessed and demonstrated the feasibility of both community health workers and innovative technology in vulnerable patient populations [31,32]. Comparatively, the Carolinas Partners model utilizes both approaches to facilitate multidisciplinary care in a targeted diabetic population with expressed readiness to change. However, the ideal multidisciplinary team composition, visit frequency and visit type to achieve medically- and cost-effective care is unknown. In today’s fee for service environment, such high level physician involvement is likely impractical. However, as value-based care delivery models evolve, demonstrating programs that are both medically- and cost-effective will become increasingly important for healthcare delivery systems working to match appropriate resources to the highest risk populations.

Of the patients who completed the study, there was a trend towards improved access to PCMH services. Based on patients’ survey data for those in the intervention group, they felt the intensive primary care services were beneficial. To adequately provide population health outreach in a vulnerable population like this with demonstrable barriers to accessing care, a coordinated, multidisciplinary approach is invaluable.

### Limitations

The small sample size of this pilot study limits the statistical analysis or drawing of conclusions regarding clinical outcomes; however, the study was intended primarily to demonstrate process feasibility. While trends in improved access and hemoglobin A1C values are present, they must be further studied. Additionally, the novel risk algorithm and readiness to change survey appear promising but warrant additional study and validation.

At the time of EMR review of eligible patients for further determination of exclusion criteria and prior to contact for screening and possible enrollment, study personnel were not blinded to the randomization, which presented an opportunity for selection bias.

Non-English speaking patients were excluded from participation due to lack of necessary resources to allow effective communication for clinical management. Thus, patients of Hispanic ethnicity who are primarily Spanish speakers were likely under-represented, considering that 28.8% of the excluded population identified as Hispanic.

Baseline access to pharmacotherapy was not assessed for either control or intervention groups prior to the study. The availability, or lack thereof, of medications for either group may serve as a confounder. A survey of access to pharmacotherapy would be helpful to elucidate this in any future study.

Due to this being an unfunded pilot study, limited study personnel availability prohibited the recruitment and management of additional participants. For example, study personnel’s future availability to participate became limited, and this precluded the team from the recruitment of additional patients, as it would not allow adequate time to complete the three-month intervention. The west region’s patients and patients of additional clinics could not be contacted due to these limitations. The process of actually enrolling patients and the ability to complete the interventional workflow were not limiting factors in determining the sample size, however.

## 6. Conclusions

In conclusion, the integration of telemedicine tools and health coaches into intensive primary care delivery offers a promising population health outreach solution that may overcome traditional barriers created by social determinants of health for vulnerable patients. Large-scale pragmatic trials are needed to assess these and other newly-emerging, innovative approaches to care delivery and population health outreach.
